# *TERT* promoter mutations are a major indicator of recurrence and death due to papillary thyroid carcinomas

**DOI:** 10.1111/cen.12999

**Published:** 2016-02-08

**Authors:** Martyn Bullock, Yan Ren, Christine O'Neill, Anthony Gill, Adam Aniss, Mark Sywak, Stan Sidhu, Leigh Delbridge, Diana Learoyd, Florent de Vathaire, Bruce G. Robinson, Roderick J. Clifton‐Bligh

**Affiliations:** ^1^ Cancer Genetics Unit Hormones and Cancer Group Kolling Institute of Medical Research Royal North Shore Hospital Sydney NSW Australia; ^2^ National Institute of Health and Medical Research (Inserm) Center for research in Epidemiology and Population Health (CESP) U1018 Radiation Epidemiology Group Villejuif France; ^3^ Gustave Roussy Institute Villejuif France; ^4^ Faculty of Medicine University of ParisXI Le Kremlin‐Bicêtre France; ^5^ University of Newcastle Sydney NSW Australia; ^6^ Department of Anatomical Pathology Royal North Shore Hospital Sydney NSW Australia; ^7^ University of Sydney Sydney NSW Australia; ^8^ University of Sydney Endocrine Surgical Unit Sydney NSW Australia; ^9^ Department of Endocrinology Royal North Shore Hospital Sydney NSW Australia

## Abstract

**Context:**

*TERT* promoter mutations have been associated with adverse prognosis in papillary thyroid carcinomas (PTCs).

**Objective:**

We investigated the association between *TERT* promoter mutations and survival from PTC.

**Design:**

Retrospective observational cohort study.

**Patients:**

Eighty consecutive patients with PTC who underwent surgery between 1990 and 2003.

**Measurements:**

*TERT* promoter was genotyped in DNA from 80 archival PTCs by Sanger sequencing. Median follow‐up was 106 months (range 1–270). Outcomes analysis was stratified according to disease and overall survival status. For each parameter, relative risk (RR) adjusted for age at first surgery and gender was estimated. Both univariate and multivariate analyses were performed using logistic regression, Kaplan–Meier survival analysis and Cox regression models.

**Results:**

PTCs from 11 patients (14%) contained either C228T or C250T *TERT* promoter mutation. *TERT* mutations were significantly associated with adverse prognostic features such as older age (*P* = 0·002), male gender (*P* = 0·01) and Stage IV disease (*P* = 0·03). Four patients died from PTC during follow‐up: 3 patients with *TERT* mutations (27%) and one without (1·5%). Disease‐related mortality rate with or without *TERT* mutations was 33·7 *vs* 1·6 per 1000 patient‐years respectively, that is 10 (95% CI = 1·0–104·1, *P* = 0·05) fold higher, after adjustment for age at first surgery and gender. The combination of *TERT* promoter mutation and *BRAF*^*V*^ ^*600E*^ significantly increased disease‐related death risk (*P* = 0·002). *TERT* mutations increased expression of a reporter gene in thyroid cells containing *BRAF*^*V*^ ^*600E*^.

**Conclusions:**

*TERT* promoter mutations are a major indicator of death due to PTCs. Conversely, absence of *TERT* mutations portends better survival.

## Background

Telomerase reactivation, either via the chromosome end‐replicating enzyme telomerase reverse transcriptase (TERT) or alternative lengthening of telomeres, is detected in up to 80% of malignant tumours.^1^, ^2^ Somatic mutations in the *TERT* promoter were first identified in melanoma^3^, ^4^ and have been observed at high frequency in multiple cancer types, including those of the thyroid, central nervous system and bladder.^5^, ^6^, ^7^ These mutations occur at two hotspots, chr5:1,295,228C>T (‘C228T’) and chr5:1,295,250C>T (‘C250T’), located, respectively, −124 and −146 bp upstream from the ATG start site. Both mutations create a putative consensus binding site (GGAA) for ETS transcription factors^3^ and correlate with increased *TERT* expression.^5^, ^6^

In thyroid cancer, *TERT* promoter mutations were initially noted to occur relatively infrequently in tumours with a better prognosis (papillary and follicular thyroid cancers) and with increasing frequency in progressively worse prognostic subtypes (i.e. poorly differentiated and anaplastic thyroid cancers).^8^, ^9^ Subsequent work demonstrated that within papillary (PTC) and follicular (FTC) thyroid cancer types, *TERT* promoter mutations were correlated to adverse prognostic features such as older age, larger tumour size and male gender.^10^, ^11^, ^12^, ^13^, ^14^ Some^10^, ^11^, ^15^ but not all^9^, ^13^, ^14^, ^16^ studies have found an interaction between the presence of *TERT* promoter mutations and oncogenic *BRAF* ^*V600E*^ in predicting recurrence. Two retrospective studies to date have found that *TERT* promoter mutations decrease survival rates in PTC and FTC.^13^, ^14^ *TERT* promoter mutations have also been identified in papillary microcarcinomas^17^ and Hurthle cell cancers^18^ but not in medullary thyroid cancers.^8^, ^9^, ^10^, ^19^ It has been possible to detect *TERT* promoter mutations in fine‐needle aspiration samples, suggesting that their pre‐operative detection may contribute to appropriate surgical assessment.^20^, ^21^, ^22^

The importance of these findings to the clinician is double: firstly, the presence of a biomarker that reliably identifies aggressive biological behaviour would justify stronger therapeutic approaches; conversely, if absence of the biomarker is associated with indolent behaviour, then such patients could be more cautiously monitored and treated accordingly.

We sought to address these issues using a retrospective cohort of PTC cases that we have been following for a median of 9 years. We tested the following hypotheses: (a) the prevalence of *TERT* promoter mutations in this Australian cohort; (b) that *TERT* promoter mutations are associated with adverse prognostic features in these PTCs; (c) that *TERT* promoter mutations are associated with disease‐free and/or overall survival; and (d) that *TERT* promoter mutations interact with *BRAF* ^*V600E*^ to influence survival.

## Materials and methods

### Study participants

This study involved 80 consecutive patients with PTC who underwent initial surgery between the years 1990 and 2003.^23^ Patients were identified from a prospectively maintained endocrine surgical database after approval from the Northern Sydney Local Health District Human Research Ethics Committee (LNR 1312‐417M).

Median clinical follow‐up was 106 months (range 1–270 months), and outcomes analyses were stratified according to disease status.

Disease‐free survival was defined as a negative clinical and radiological examination (i.e. high‐resolution neck ultrasonography and/or whole‐body radioactive iodine scan) in the presence of a serum thyroglobulin level <1 μg/l and undetectable thyroglobulin antibodies (<40 kIU/l). Deceased patients were classified as a disease‐related death or, if their death was unrelated to PTC, classified according to their disease status at the time of death.^23^

Nodal recurrence was defined as reappearance of PTC within the neck after completion of initial treatment (total thyroidectomy and radioactive iodine remnant ablation). Tumour recurrence was confirmed on the basis of a positive fine‐needle aspiration cytology, abnormal sonographic appearance or positive radioiodine imaging.^23^

### Tissue samples

FFPE tumour blocks from initial surgery for each patient were obtained from the archives of the Department of Anatomical Pathology at the Royal North Shore Hospital. Fresh sections were cut from archived blocks and independently reviewed by a single pathologist to confirm the diagnosis.^24^ The single paraffin block with the largest dimension of tumour regardless of the ratio of neoplastic to non‐neoplastic tissue was chosen for genomic DNA extraction for subsequent Sanger sequencing.

### Genomic DNA extraction and PCR

From each paraffin block confirmed to contain tumour, a ribbon of 10 × 10 μm sections was placed in an Eppendorf container with care being taken to prevent tissue cross‐contamination. Genomic DNA was extracted from the FFPE sections using a QIAamp DNA FFPE Tissue Kit; (Qiagen, Hilden, Germany), according to the manufacturer's instructions. *TERT* promoter was amplified with HotStartTaq Polymerase Plus (Qiagen) and using the nested PCR methodology utilized by Horn *et al*.^3^ Briefly, the first‐round PCR amplicon was amplified using forward 5′‐ACGAACGTGGCCAGCGGCAG and reverse 5′‐CTGGCGTCCCTGCACCCTGG primers with an annealing temperature of 62 °C. Then, the second‐round PCR amplicon was amplified using forward 5′‐CAGCGCTGCCTGAAACTC and reverse 5′‐GTCCTGCCCCTTCACCTT primers with an annealing temperature of 55 °C. The reaction for both rounds of PCR contained Qiagen's 20% Q‐solution. All PCR runs included a standard no DNA template control, which was negative in all cases.

### Sanger sequencing

PCR products were purified using Wizard SV Gel and PCR Clean‐up System (Promega, Sydney, NSW, Australia) according to the manufacturer's instructions. Each sample was sequenced using forward and reverse primers on an ABI PRISM 3700 platform (Applied Biosystems, Foster City, CA, USA) (service provided by Australian Genome Research Facility, Sydney, Australia). All samples were genotyped at least twice by two different scientists who utilized their own PCR reagents. Positive controls were included with each sequencing run: normal thyroid (wild type)‐ and cancer cell (e.g. the C228T‐positive SW1736 cell‐line)‐derived genomic DNA that yielded the expected *TERT* promoter sequence in each case.

### Cell culture studies

A region of the *TERT* promoter encompassing −391 to +83 bp (relative to the TSS) was cloned into pGL3‐basic (Promega) and termed TERT‐LUC. Site‐directed mutagenesis was then performed to generate mutations corresponding to C228T (TERT228‐LUC) and C250T (TERT250‐LUC). These vectors were transfected in triplicate into SW1736 cells (derived from an anaplastic cancer containing *BRAF* ^*V600E*^ but not *RAS* mutations and exhibiting low p53 expression although without *TP53* mutation^9^, ^25^) and treated with either vehicle or the MEK inhibitor U0126. After 48 h, cells were lysed and luciferase activity was measured as previously described.^26^ *Renilla* luciferase was used as an internal transfection control to normalize the data.

### Statistical analyses

To analyse the association between clinicopathological and molecular factors with *TERT* promoter mutations in PTCs, we used logistic regression, Fisher's exact test, Cochran–Mantel–Haenszel test stratified for age at first surgery (as a categorical variable) and gender, considering *TERT* promoter mutations as the dependent variable, and each clinicopathological or molecular factor involved in the univariate or multivariate model as independent variables. Both univariate and multivariate survival analyses were performed using Cox regression model and for each variable adjusting for age at first surgery (as a continuous variable) and gender. In univariate analyses, the variables whose p‐values were <0·05 were retained in multivariate analyses. Tumour size was included in the model as a continuous variable as only a few PTC samples had microscopic deposits (i.e. <10 mm). We used Kaplan–Meier survival curves to present either overall survival (considering only disease‐related deaths) or disease‐free survival (where ‘disease’ was defined as persistent or recurrent nodal or distant metastases, or disease‐related death), according to different clinicopathological and molecular features, such as age at first surgery, gender, tumour size, stage, *BRAF* ^*V600E*^ and *TERT* (C228T or C250T) via the logrank test. Statistical significance was set at *P* < 0·05. Statistical analyses were performed using SAS software version 9·3 (SAS Institute, Inc., Cary, NC, USA) and StatXact version 11.0.0 Cytel Inc (Cambridge, MA, USA).

## Results

The study cohort has been previously described^23^; of the 100 subjects in our previous report, 20 FFPE samples failed to amplify the *TERT* promoter and were excluded from this analysis. Our cohort therefore consisted of 80 patients (14 men) with mean age 47·3 years at first surgery and a median duration of follow‐up of 106 months (range 1–270). Overall, 59 (74%) were disease‐free at the end of the follow‐up period, 9 had persistent or recurrent disease (11%), 8 had died of PTC‐unrelated causes (10%), and 4 (5%) had died from PTC. If those patients who had died from unrelated causes were reclassified according to their disease status at the time of death, then 66 of 80 (83%) were disease‐free whereas 14 of 80 (18%) had persistent or recurrent disease or had died from PTC. Baseline characteristics of the cohort according to the presence or absence of *TERT* promoter mutations are presented in Table 1. A total of 11 of 80 patients (14%) had PTCs containing *TERT* promoter mutations: 8 had the C228T mutation and 4 had the C250T mutation (one tumour had both mutations). *TERT* promoter mutations were significantly more frequent in PTC with adverse prognostic features such as older age (*P* = 0·002; Fisher's exact test), male gender (*P* = 0·01; chi‐squared test, two‐sided), and Stage IV disease (*P* = 0·03, Cochran–Mantel–Haenszel test, two‐sided). No significant heterogeneity according to age at the first surgery and gender categories was evident for an association between *TERT* promoter mutations and tumour size (*P*‐value for heterogeneity of odds‐ratio = 1), that is the lack of association between *TERT* promoter mutations and tumour size (*P* = 0·8, Cochran–Mantel–Haenszel test) may be considered as true whatever age at first surgery and gender. *BRAF* ^*V600E*^ was present in 58 PTCs (73%). Eight patients (10%) had both *TERT* promoter mutation and *BRAF* ^*V600E*^ (Table 1).

**Table 1:** Summary of clinicopathological or molecular associations with *TERT* promoter mutations in papillary thyroid cancers

	PTC N	TERT (C228T or C250T) N (%)	Relative risk (95% CI)	*P*‐Value
Total number	80	11 (13·8)		
Age at first surgery^a^				
Mean ± SD	47·3 ± 17·6	62·6 ± 15·3		0·002
≤45	43	1 (2·3)	1 (Referent)	
>45	37	10 (27·0)	15·2 (1·9–674·6)	
Gender				
Male	14	5 (35·7)	1 (Referent)	0·01
Female	66	6 (9·1)	0·2 (0·05–0·7)	
Histological subtype^b^^,^ c				
Classical	58	8 (13·8)	1 (referent)	0·2
Follicular	16	0 (0·0)	NCR	
Tall cell	5	3 (60·0)	6·6 (0·8–53·9)	
Tumour size (cm)^b^^,^ c				
Mean ± SD	1·1 ± 0·5	1·3 ± 0·6		0·8
<1	6	1 (16·7)	3·1 (0·2–40·4)	
1 – 4	63	6 (9·5)	1 (Referent)	
>4	11	4 (36·4)	2·2 (0·4–12·2)	
Lymph nodes with cancer^b^^,^ c				
No	51	5 (9·8)	1 (Referent)	0·1
Yes	29	6 (20·7)	3·4 (0·8–15·6)	
Stage^b^^,^ c				
I+II+III	73	7 (9·6)	1 (Referent)	0·03
IV	7	4 (57·1)	9·1 (1·3–64·4)	
BRAF status^b^^,^ c				
No	22	3 (13·6)	1 (Referent)	0·3
Yes	58	8 (13·8)	2·7 (0·3–23·3)	

NCR, no convergence reached.

a Fisher's exact test.

b Adjusted for age at the first surgery as a categorical variable (≤45 or >45) and gender using logistic regression.

c Cochran–Mantel–Haenszel test stratified for age at the first surgery on class and gender.

Disease‐related mortality according to *TERT* promoter mutations is presented in Table 2. Four patients (5%) died from their PTC during follow‐up (mean 50 months, median 42 months, range 2–113 months): 3 of 11 subjects (27%) with *TERT* promoter mutations died, whereas only 1 of 69 (1·4%) with wild‐type *TERT* sequence died. Hazard ratio for disease‐related death associated with *TERT* promoter mutations was 10·0 (95% confidence intervals = 1·0–104·1, *P* = 0·05) after adjusting for age at the first surgery as a categorical variable (≤45 or >45) and gender.

**Table 2:** Papillary thyroid cancer‐related mortality and hazard ratios for patients with *TERT*‐mutated *vs TERT* wild‐type sequence

Disease‐related mortality, *n* (%)			Disease‐related mortality rate (Deaths per 1000 Person‐Years) (95% CI)		Hazard ratio (95% CI)			
Overall	TERT Mutated	TERT a wt	TERT Mutated	TERT a wt	Unadjusted	*P*‐Value^b^	Adjusted^c^	*P*‐Value^b^
4/80 (5·0)	3/11 (27·3)	1/69 (1·5)	33·7 (8·1–532·0)	1·6 (0·0–14·1)	19·7 (2·0− 189·5)	0·0003	10·0 (1·0–104·1)	0·05

a TERT wt: TERT wild‐type sequence.

b Exact Logrank test.

c Adjusted for age at the first surgery as a categorical variable (≤45 or >45) and gender using Cox regression.

**Figure 1:**
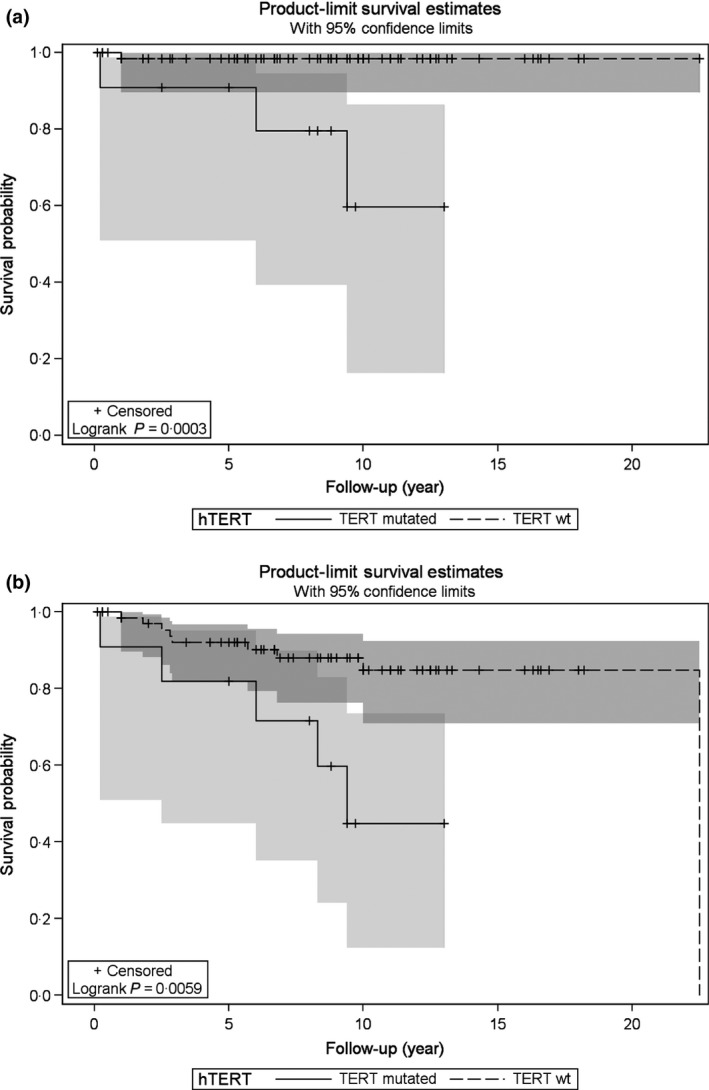
Kaplan–Meier Survival Curves of PTC‐Overall Survival (a) and PTC‐Disease‐free Survival (b) by *TERT* Status. Survival of subjects with *TERT* mutations (either C228T or C250T) is shown by the *continuous* line whereas survival of subjects with wild‐type *TERT* sequence is shown by the *dotted* line.

The presence of *TERT* promoter mutations (C228T or C250T) (logrank *P* = 0·0003) was significantly associated with decreased overall survival due to PTC‐related death (Fig. 1a) and also with decreased disease‐free survival (Fig. 1b).

Of 20 patients with either Stage III or IV disease at presentation (13 Stage III, 7 Stage IV), *TERT* promoter mutations were present in 7, of whom 3 died (43%) compared with one death in 13 patients (8%) with wild‐type *TERT* promoter sequence.

Examining the possible interaction between *TERT* promoter mutations and *BRAF* ^*V600E*^, the mortality rate for subjects whose tumours were wild type for both genotypes was 0/18; for *TERT‐*mutated but wild‐type *BRAF* 0/3; for *BRAF* ^*V600E*^ but wild‐type *TERT* 1/51 (2%); and for *TERT* mutated and *BRAF* ^*V600E*^ together mortality was 3/8 (38%). The relative risk of disease‐related death in patients with both *TERT* mutated and *BRAF* ^*V600E*^ mutated together was 29·4 (Cox’ model with profile‐likelihood 95% confidence limits = 3·8–594·6), as compared to the other patients (*P* = 0·002) (Table 3).

**Table 3:** Hazard ratios of disease‐related death associated with *TERT* promoter mutations and *BRAF* ^*V600E*^

	Presence *n* (%)	Hazard ratio (95% CI)^e^	*P*‐Value^f^
*TERT* wt & *BRAF* wt^a^	0/18 (0)	NCR	NCR
*TERT* mutated & *BRAF* wt^b^	0/3 (0)	NCR	NCR
*TERT* wt & *BRAF* mutated^c^	1/51 (2·0)	0·2 (0·01–1·4)	0·08
*TERT* mutated & *BRAF* mutated^d^	3/8 (37·5)	29·4 (3·8–594·6)	0·002

NCR, no convergence reached.

a *TERT* wild‐type sequence and *BRAF* wild‐type sequence compared with the other 3 groups (b+c+d).

b *TERT*‐mutated but *BRAF* wild‐type sequence compared with the other 3 groups (a+c+d).

c *TERT* wild‐type sequence but *BRAF* mutated compared with the other 3 groups (a+b+d).

d *TERT* mutated and *BRAF* mutated compared with the other 3 groups (a+b+c).

e Cox' model with profile‐likelihood confidence limits.

f Logrank exact test.

**Figure 2:**
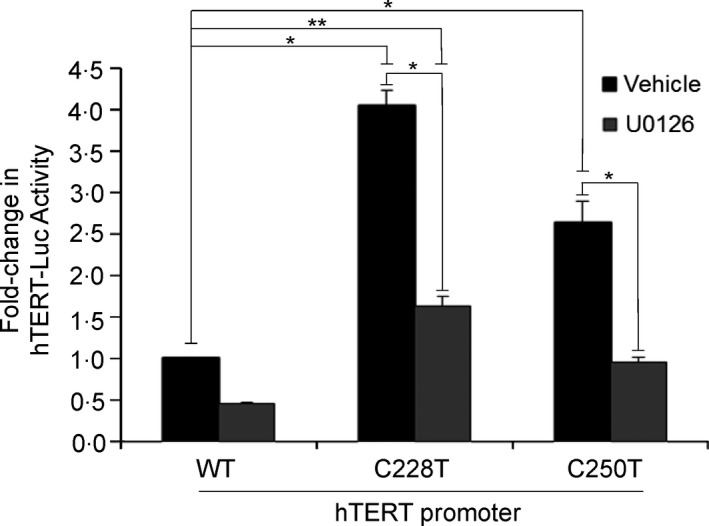
The effect of *TERT* promoter mutations on reporter gene activity. SW1736 cells were transfected (in triplicate) with TERT‐LUC, TERTC228T‐LUC or TERTC250T‐LUC as shown and treated with vehicle or the MEK inhibitor U0126. Luciferase activity was measured in cell lysates 48 h after transfection and normalised for *Renilla* luciferase as a transfection control. Data shown are mean ± SD from three experiments. *P < 0·01.

We therefore explored a possible interaction *in vitro* using reporter gene containing either the wild‐type or mutated *TERT* promoter. As shown in Fig. 2, *TERT* promoter constructs containing either C228T or C250T mutations exhibited increased transcriptional activation when transfected in SW1736 cells (endogenously containing *BRAF* ^*V600E*^): TERTC228T‐Luc showed 4·05 ± 0·19‐fold increase (*P* = 0·001), and TERTC250T‐LUC showed 2·6 ± 0·26‐fold increase (*P* = 0·008) relative to TERT‐LUC. The addition of MEK inhibitor U0126 resulted in significant reduction of promoter activity for all constructs (Fig. 2).

We also considered the association between *TERT* promoter mutations and the composite end‐point of disease persistence or recurrence, or disease‐related death. Fourteen patients were not disease‐free at the end of follow‐up (Table 4). In univariate analysis, *TERT* promoter mutations (HR = 4·3, 95% CI = 1·4–13·3, *P* = 0·01), older age at initial surgery (HR = 1·05, 95% CI = 1·01–1·08, *P* = 0·01), larger tumour size (HR = 1·04, 95% CI = 1·01–1·06, *P* = 0·01) and Stage IV disease (HR = 14·0, 95% CI = 4·4–43·9, *P* < 0·0001) were all significantly associated with a lower disease‐free survival. However, in multivariate analysis, only Stage IV disease (HR = 16·5, 95% CI = 2·9–93·5, *P* = 0·002) remained significant (Table 4).

**Table 4:** Hazard ratios for persistent or recurrent disease or disease‐related death

	Presence (*n* = 14)	Univariate analysis		Multivariate analysis^a^	
		Hazard ratio (95% CI)	*P*‐Value	Hazard ratio (95% CI)	*P*‐Value
Age at the first surgery^b^					
Gender	14	1·05 (1·01–1·08)	0·01	1·00 (0·96–1·04)	0·9
Male	3	1 (Referent)	0·5	1 (Referent)	0·5
Female	11	0·6 (0·2–2·3)		0·6 (0·1–2·5)	
Tumour Size (cm) c	14	1·04 (1·01–1·06)	0·01	1·0 (1·0–1·1)	0·1
Stage					
I, II, III	8	1 (Referent)	<0·0001	1 (Referent)	0·002
IV	6	14·0 (4·4–43·9)		16·5 (2·9–93·5)	
TERT (C228T or C250T)					
No	9	1 (Referent)	0·01	1 (Referent)	0·6
Yes	5	4·3 (1·4–13·3)		0·6 (0·1–3·1)	

a Adjusted for age at the first surgery as a continuous variable and gender using Cox regression.

b Age at the first surgery was included in the model as a continuous variable.

c Tumour size was included in the model as a continuous variable.

## Discussion

In the current study, we observed that *TERT* promoter mutations (either C228T or C250T) were significantly associated with adverse prognostic features such as older age, male gender and Stage IV disease. *TERT* promoter mutations were significantly associated with disease‐related mortality (33·7 deaths per 1000 patient‐years compared with 1·6 per 1000 patient‐years for wild‐type *TERT*) and the hazard ratio for disease‐related death was 10·0 (95% CI = 1·0–104·1, *P* = 0·05) after adjustment for age at the first surgery and gender. In Stage III or IV disease, mortality was 43% in patients with *TERT* mutations compared with 8% in those with wild‐type *TERT*. The presence of both a *TERT* promoter mutation and *BRAF* ^V600E^ significantly increased disease‐related death risk (*P* = 0·002) compared with *TERT* wild‐type sequence and *BRAF* wild‐type sequence, *TERT*‐mutated but *BRAF* wild‐type sequence, or *TERT* wild‐type sequence but *BRAF* mutated. We also found *in vitro* evidence that *TERT* mutations increased transcriptional activity of a reporter gene in thyroid cells containing *BRAF* ^*V600E*^ and that this effect was reversed by treatment with a MEK inhibitor.

Our prevalence of 14% for *TERT* mutations in PTC is consistent with other reports; globally, an average of 11·5% PTCs harbour *TERT* mutations (Table S1). The associations with older age, male gender and Stage IV disease are also consistent with previous reports.^8^, ^9^, ^10^, ^11^, ^12^, ^13^, ^14^ In our study, the presence of *TERT* mutations was associated with the composite outcome of disease recurrence, persistence or death in univariate analysis, but was no longer significant after accounting for tumour size and Stage IV disease. In larger studies,^11^, ^13^ *TERT* promoter mutations have remained significantly associated with disease persistence in multivariate analyses.

Our data are broadly consistent with two other studies that have reported *TERT*‐associated mortality data to date^13^, ^14^ (Table S1). Melo and colleagues^13^ studied 332 patients with PTC, and found that *TERT* promoter mutations were present in 25 (7·5%); after a mean of 7·8 year follow‐up, 2 of 19 patients with *TERT* promoter mutations had died (10·5%) compared to 3 of 265 patients (1·1%) with wild‐type *TERT* promoter sequence.^13^ Mortality rate was very similar in our study (33·7 *vs* 1·6 per 1000 person‐years for PTCs with and without *TERT* promoter mutations, respectively) compared with that of Melo *et al*. (13·64 *vs* 1·36 deaths per 1000 person‐years).^13^ In a study of high‐risk (i.e. already persistent/recurrent) PTC, George and colleagues^14^ found the C228T *TERT* promoter mutation in 77 of 242 cases (31·8%); after median follow‐up of 112 months, 19 of 77 patients with *TERT* mutation had died (24·7%) compared to 10 of 165 (6·1%) with wild‐type *TERT*.

Our *in vitro* data indicating that *TERT* promoter mutations increased reporter gene expression by 2‐ to 4‐fold in SW1736 thyroid cells are consistent with studies that show similar stimulation of promoter activity in melanoma, urothelial and hepatoma cell lines^4^, ^27^ and in BCPAP and K2 thyroid cell lines.^14^ We then examined whether MEK inhibition could rescue the effect of these mutations on promoter activity, our rationale being that *TERT* promoter mutations are activating by virtue of creating new binding sites for MAPK‐responsive transcription factors.^3^ We chose SW1736 cells for this study, because they are known to endogenously contain *BRAF* ^*V600E*^.^9^ We found that treatment with the MEK inhibitor U0126 did significantly reduce the activity of reporter genes containing either *TERT* C228T or C250T mutations. This observation provides a basis for potential treatment of *TERT‐*mutated thyroid cancers using inhibitors of the MAPK pathway.

It is well known that some patients with metastatic PTC will have a very indolent course even without aggressive treatment approaches.^28^ We found that in patients presenting with Stage III or IV disease, the presence of *TERT* mutations increased mortality but conversely, the absence of *TERT* mutations was associated with generally good prognosis (>70% survival after 8 years). If corroborated in larger studies or by meta‐analysis, this would be clinically important: patients with *TERT‐*mutated advanced disease could be triaged early to aggressive treatment with kinase inhibitors whereas patients with *TERT* wild‐type disease could be treated more cautiously.

In conclusion, *TERT* promoter mutations (C228T or C250T) are a major indicator of death due to PTC. Meta‐analysis will be needed to confirm that *TERT* promoter mutations interact with *BRAF* ^*V600E*^ to increase PTC‐related mortality.

## Funding

MB and RCB were supported by NHMRC project grant 1061941. YR is supported by the Fondation de France and a grant from 2014 Inserm – University of Sydney Research Mobility Funding Scheme.

## Conflict of interest

All authors have declared that there is no conflict of interest exists in relation to this article.

## Supporting information


**Table S1.** *TERT* promoter mutations in thyroid cancer samples.
