# Supplementary material for: TERT promoter mutations are a major indicator of recurrence and death due to papillary thyroid carcinomas
Source: Clin Endocrinol (Oxf). 2016 Feb 8;85(2):283–90. doi: 10.1111/cen.12999 (PMC5683578; doi:10.1111/cen.12999)
Supplement: Supplementary file 1 — Table S1. TERT promoter mutations in thyroid cancer samples. [file CEN-85-283-s001.docx]

**Supplementary Table. *TERT* promoter mutations in thyroid cancer samples**

**A. Prevalence**

**First Author Ref Country Benign PTC FTC PDTC ATC MTC**

Vinagre (5) Portugal 0/81 13/169 9/64 3/14 2/16 0/28

Liu (8) USA 0/85 30/257 11/79 3/8 25/54 0/16

Landa (9) I (USA) - 8/29 - 30/58 10/20 -

II (Japan) - 10/51 - - - -

Liu (10) China 0/44 46/408 8/22 - - -

Xing (11) USA - 61/507 - - - -

Wang (12) Sweden - - 9/52 - - -

Melo (13) Portugal - 25/339 12/70 9/31 12/36

Muzza (15) Italy 0/6 22/182 8/58 - - 0/14

Gandolfi (22) Italy - 21/121

Current study Australia - 11/80 - - - -

Total (PTC) 247/2143 (11.5%)

**B. Survival**

First Author Ref Country Follow-up HR (CI) Mortality (/1000 py)

*TERT*mut vs *TERT*wt *TERT*mut vs *TERT*wt

adjusted for age, gender

Melo (13) Portugal (PTC) 7.8±5.8 y 23.81 (1.36-416.76) 13.64 vs 1.36

(DTC) 10.35 (2.01-53.24) 21.17vs 1.17

Current study Australia (PTC) 8.3 ±4.8 y 10.0 (1.0-104.1) 33.7 vs 1.6

George et al (14) studied high-risk PTC and found a risk ratio of death associated with *TERT* promoter mutation of 4.46 (2.59-7.67).
